# Nucleoporin 153 Arrests the Nuclear Import of Hepatitis B Virus Capsids in the Nuclear Basket

**DOI:** 10.1371/journal.ppat.1000741

**Published:** 2010-01-29

**Authors:** André Schmitz, Alexandra Schwarz, Michael Foss, Lixin Zhou, Birgit Rabe, Julia Hoellenriegel, Miriam Stoeber, Nelly Panté, Michael Kann

**Affiliations:** 1 Institute of Medical Virology, Justus Liebig University, Giessen, Germany; 2 UMR-CNRS 5234 MCMP, Université Bordeaux 2, Bordeaux, France; 3 Department of Zoology, University of British Columbia, Vancouver, British Columbia, Canada; Fox Chase Cancer Center, United States of America

## Abstract

Virtually all DNA viruses including hepatitis B viruses (HBV) replicate their genome inside the nucleus. In non-dividing cells, the genome has to pass through the nuclear pore complexes (NPCs) by the aid of nuclear transport receptors as e.g. importin β (karyopherin). Most viruses release their genome in the cytoplasm or at the cytosolic face of the NPC, as the diameter of their capsids exceeds the size of the NPC. The DNA genome of HBV is derived from reverse transcription of an RNA pregenome. Genome maturation occurs in cytosolic capsids and progeny capsids can deliver the genome into the nucleus causing nuclear genome amplification. The karyophilic capsids are small enough to pass the NPC, but nuclear entry of capsids with an immature genome is halted in the nuclear basket on the nuclear side of the NPC, and the genome remains encapsidated. In contrast, capsids with a mature genome enter the basket and consequently liberate the genome. Investigating the difference between immature and mature capsids, we found that mature capsids had to disintegrate in order to leave the nuclear basket. The arrest of a karyophilic cargo at the nuclear pore is a rare phenomenon, which has been described for only very few cellular proteins participating in nuclear entry. We analyzed the interactions causing HBV capsid retention. By pull-down assays and partial siRNA depletion, we showed that HBV capsids directly interact with nucleoporin 153 (Nup153), an essential protein of the nuclear basket which participates in nuclear transport via importin β. The binding sites of importin β and capsids were shown to overlap but capsid binding was 150-fold stronger. *In cellulo* experiments using digitonin-permeabilized cells confirmed the interference between capsid binding and nuclear import by importin β. Collectively, our findings describe a unique nuclear import strategy not only for viruses but for all karyophilic cargos.

## Author Summary

Viral capsids facilitate protection of the enclosed viral genome and participate in the intracellular transport of the genome. At the site of replication capsids have to release the genome. The particular factors triggering genome liberation are not well understood. Like other karyophilic cargos, hepatitis B virus (HBV) capsids are transported through the nuclear pore using nuclear transport receptors of the importin ß superfamily. Unlike physiological cargos, HBV capsids become arrested within the nuclear basket, which is a filamentous structure on the nuclear side of the nuclear pore. Asking which interaction causes this unique strategy, we found that the capsids bind to a protein of the basket periphery, nucleoporin 153 (Nup153). The findings were confirmed *in situ* using digitonin-permeabilized cells that support physiological genome delivery into the nucleus. We observed that HBV capsids bound to Nup153 irrespective of the maturation of the encapsidated genome. But while capsids with an immature genome remained in arrested state, capsids with a mature genome disassembled and released their DNA.

## Introduction

Most DNA viruses depend on nuclear host factors for their replication. Viruses infecting non-dividing cells have to pass the nuclear envelope through the nuclear pore complexes (NPCs). The NPC is large proteinaceous structure of ∼30 different proteins called nucleoporins (Nups). Due to the eight fold rotational symmetry of the NPC each Nup is present in 8–48 copies, forming a complex of ∼125 MDa. On the cytoplasmic face of the NPC eight fibers extrude from a central ring-like framework, which is embedded in the nuclear envelope. This ring forms openings in the nuclear envelope allowing translocation of cargos with a diameter up to 39 nm [1]. On the karyoplasmic face of the NPC 8 fibers form the cage-like structure of the nuclear basket (reviewed by [2]).

NPCs regulate the traffic of proteins and nucleic acids into and out of the nucleus (reviewed by [3]). While small molecules may diffuse between cytoplasm and nucleus karyophilic macromolecules are transported in a complex with soluble nuclear import receptors. It is estimated that ∼1000 transport complexes pass each NPC per second [4].

The best characterized transport receptors belong to the importin (karyopherin) β superfamily, comprising importin β, transportin 1, 2, transportin SR and exportins. Nuclear import is initiated by binding of the receptors to a signal on the surface of the karyophilic cargo. There is a variety of signals as e.g. M9 domains, interacting with transportins, importin β binding domains and “classical” nuclear localization signals (NLS), which bind to importin β via the adapter molecule importin α.

The driving force of nuclear import and export is a gradient of the small GTPase Ran in its GTP-bound form across the nuclear envelope. RanGTP is enriched in the karyoplasm, where it interacts with the transport receptors of the import complex, leading to its dissociation. While the cargo diffuses deeper into the karyoplasm, the RanGTP-receptor complex is exported into the cytoplasm where it dissociates.

A key component of nuclear import is Nup153 to which the import complex of cargo and importin(s) binds and subsequently dissociates. Nup153 is a 1445 amino acid (aa) protein of the nuclear basket [5], which comprises three domains (reviewed by [6]). The N terminus (aa 1–670) at the nuclear ring of the NPC contains an NPC targeting domain and an RNA binding domain. The N terminus interacts with other proteins of the NPC as Nup107 and Tpr and is required for proper NPC architecture [5],[7],[8]. The zinc finger region (aa 650–880) at the distal ring of the NPC facilitates interactions with Ran. The ∼30 FXFG repeat-containing C terminus is part of the hydrophobic meshwork that forms the “sieve” through which karyophilic cargos have to pass [9]. The participation of Nup153 in vital cellular processes makes it difficult to analyze its functions without interfering with cell viability [10].

Viruses with a nuclear phase in their life cycle make use of this import machinery. Best characterized are adeno-, herpes, influenza and the human immune deficiency virus. The latter two viruses disassemble in the cytoplasm and release the genome in complex with karyophilic viral proteins. These complexes fall below the limit of the NPC diameter and pass the pore like cellular macromolecules (reviewed by [11]) or by transiently interacting with Nups [12] In contrast the genomes of adenoviruses and herpes viruses remain encapsidated within their capsids. Their diameters of 90 and 120 nm exceed the diameter of the nuclear pore. Adenoviruses bind to the cytoplasmic face of the NPC where they disassemble. Genome translocation through the pore involving viral and cellular karyophilic proteins is not well understood [13],[14],[15],[16]. Herpes virus capsids also bind to the exterior of the NPC using importin β [17] but they open at the penton facing the NPC and inject the DNA through the pore. For both viruses the trigger of genome release is unknown.

Similar investigations have been performed in digitonin-permeabilized cells on capsids of the medically important hepatitis B virus (HBV) [18],[19]. Hepatitis B is endemic in large parts of the world. Approximately 350 million people are chronically infected, accounting for 1 million deaths per year. HBV is an enveloped virus comprising an icosaedral capsid, which contains the partially double stranded DNA genome (relaxed circular, rcDNA). The capsid exists in T = 4 (major form) and T = 3 symmetry [20], composed of 240 or 180 copies of the core protein, respectively. The two forms differ in diameter (36 and 32 nm) but functional differences are not known. Entry of the virus into the hepatocyte is not well understood due to low efficiency of the available cell culture systems for HBV infection [21]. Circumstantial evidence however suggests that the capsid enters the cytosol after fusion of the viral envelope with a cellular membrane [22]. In fact lipofection of capsids, which by-passes the rate-limiting natural entry causes productive HBV “infection” of hepatoma cells with *in vivo*-like efficiency [23]. Like other DNA viruses (with the exception of baculoviruses [24]) HBV capsids are transported towards the NPCs using the microtubule transport system [23].

HBV is a pararetrovirus replicating via an RNA pregenome that is transcribed from the nuclear covalently-closed circular form of the viral genome. Consequently the viral rcDNA has to enter the nucleoplasm upon infection where the rcDNA is converted to the covalently-closed circular form. To allow access of the unknown cellular DNA repair factors the viral genome has to be liberated from the capsid either prior to, during or after transport through the NPC. Transport and genome release are obviously highly efficient and well-coordinated, since ∼80% of virions are infection-competent *in vivo* [25].

After export to the cytoplasm, the RNA pregenome is translated to core protein and the viral polymerase which binds *in situ* to an encapsidation signal on the pregenome. This complex is specifically encapsidated within the assembling core protein (reviewed by [26]), which forms an immature capsid (Immat-C). The polymerase converts the RNA into rcDNA, which is found in mature capsids (Mat-C). It is worth noting that genome maturation occurs exclusively inside the capsid and is a prerequisite for envelopment of the capsids by the surface proteins for virion formation [27].

Core proteins assemble spontaneously to HBV capsids, e. g. upon expression in *E. coli*. The first 140 aa of the core protein are essential for assembly and exhibit an ordered structure. Within a resolution limit of 16 Å *E. coli*-expressed capsids show the same morphology as virion-derived capsids or capsids from infected cells [28]. The arginine-rich C-terminus (aa 141–185) is flexible [29] and exhibits phosphorylation sites for a cellular protein kinase, which has not yet been unequivocally identified. In *E. coli*-expressed capsids, which contain unspecific *E. coli* RNA and which are not phosphorylated, the C terminus is localized within the lumen of the capsid [30].

Some steps of genome maturation depend upon core protein phosphorylation, notably pregenome encapsidation and plus strand DNA synthesis [31],[32]. Both phosphorylation and genome maturation lead to translocation of the core protein C termini, harboring a NLS, to the exterior of the capsids [19]. Consequently, all HBV capsids that have undergone some degree of genome maturation or phosphorylation bind to importin α/β [18] and can be found in the nuclear basket [1],[19]. Early in infection when sufficient amounts of surface proteins have not yet been synthesized, nuclear entry of progeny genomes increases the number of nuclear HBV DNA copies [33], which is generally low but can reach numbers of up to 491 per cell in HBV infected patient [34].

Using Digitonin-permeabilized cells – a frequently used system for analysis of nuclear import [35] – it was however shown that Immat-C and *in vitro* phosphorylated capsids synthesized in *E. coli* (P-rC) failed to exit the nuclear basket and thus do not diffuse deeper into the karyoplasm. In contrast, adding Mat-C to the cells led to the presence of intranuclear capsids and genome release [18],[19].

The import strategy of HBV capsids seems thus to follow an entirely different strategy than has been shown for other viruses. In particular the arrest of Immat-C in the basket is unique. To date the only examples for cargos with an aborted nuclear import reaction are some Nups that become incorporated into the NPC after cell division and the protein Ubc9, which partly associates with Nup358 on the cytosolic fibers of the NPC.

Evidently, the viral capsids have to interact with the NPCs upon initial infection but during establishment of the infection as well, at which time the nuclear viral DNA becomes amplified. Due to the incomplete understanding of HBV transport and disassembly, the unique strategy and the medical importance of HBV, we evaluated the molecular background of the capsid arrest in the NPC basket. Using *in vitro* and *in cellulo* approaches we identified the cellular interaction partner and identified the underlying mechanism responsible for the selective release of mature viral genomes. Collectively, these findings lead to a model of a multi-step, maturation-regulated nuclear entry of the HBV genome.

## Results

### Co-immune precipitation of nuclear proteins

Abortion of a nuclear import reaction within the nuclear pore must be based on a direct or indirect interaction with proteins of the NPC. The recently observed arrest of Immat-C and P-rC in the nuclear basket thus most likely involves an association with a Nup on the karyoplasmic face of the NPC. According to the current view on the architecture of the NPC candidates would include Nup50, 54, 58, 62, 93, 96, 98, 107, 133, 153, 160, Rae1, Seh1, Sec13 PBC68, and Tpr (summarized by [36]).

First, we wanted to identify the NPC proteins that are co-precipitated by Immat-C using a nuclear extract of rat liver nuclei. The NPC is composed of tightly interacting mostly hydrophobic proteins requiring denaturating conditions or harsh detergents for separation. As this treatment unfolds proteins we first tested whether refolding restored biological functions. For this purpose, we investigated the importin β binding ability since it is one of the essential functions of Nup153. Affinity of Nup153 to importin β was shown to be stronger than to other nucleoporins (Nup62, Nup214, Nup358 [37]).

To test our experimental system, pull-down was performed by incubating recombinant functional importin β with the extract followed by binding of the nucleoporins to solid-phase bound antibody mAb414. This antibody binds preferentially to the FXFG-repeat containing nucleoporins of vertebrates as e.g. Nup62, Nup153, Nup214 and Nup358 [38],[39] with different efficiency.

**Figure 1:**
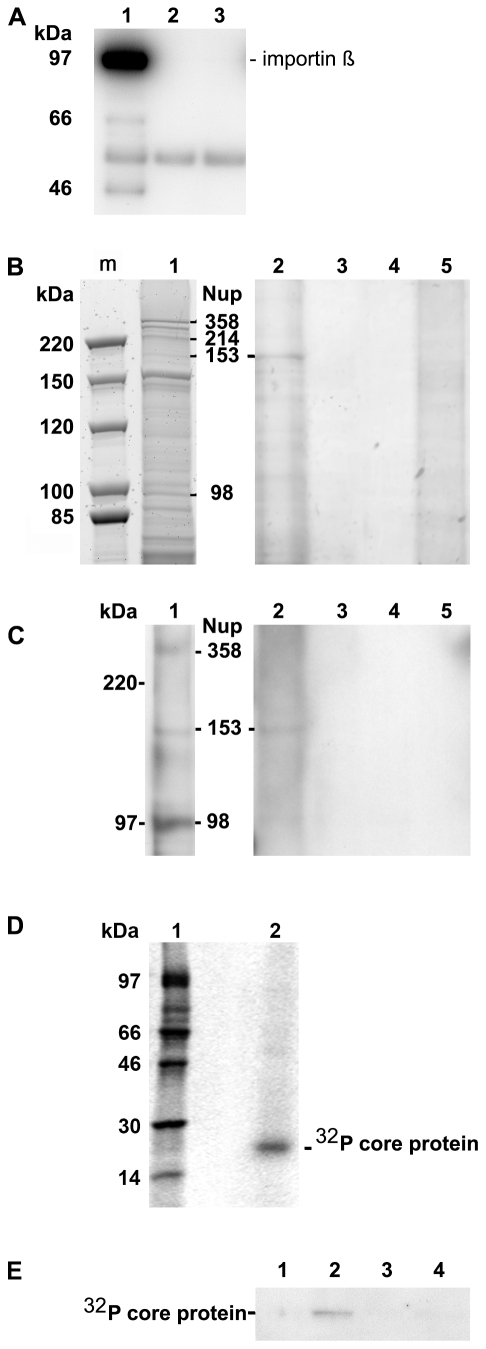
Co-immune precipitation of Nup153 from rat nuclear extracts with Immat-C. **A.** Importin β binding of Nups after renaturation. Nuclear extract was incubated with importin β and FXFG repeat-containing Nups were precipitated using mAb414-coated biomagnetic beads. Panel A depicts an immune blot using an anti-importin β antibody showing that importin β was co-precipitated by the nucleoporins. 1: mAB414-coated beads + nuclear extract+importin β. 2: mAB414-coated beads without nuclear extract + importin β. 3: Uncoated beads+nuclear extract + importin β. The 54 kDa band present in all lanes represents the heavy chain of the antibodies. The bands at 66 and 47 kDa in lane 1 are most likely degradation products of Imp β. **B.** Protein co-precipitation by capsids from nuclear extract. Sypro Red stain after SDS PAGE. m: marker. The MW is listed on the left. 1: nuclear extract without precipitation. The migration of different nucleoporins is indicated on the right. 2: Co-immune precipitation of nuclear extract with Immat-C bound to anti capsid antibody-coated biomagnetic beads. 3: Negative control without nuclear extract. 4: Beads only. 5: Co-immune precipitation of nuclear extract without Immat-C by anti capsid antibody-coated biomagnetic beads. The stain shows a precipitation of a protein with an apparent MW of ∼180 kDa. **C.** Immune blot of the nuclear protein(s), which were co-precipitated by Immat-C. Immune detection was performed using mAb414. 1–5 as in Figure 1B. The blot shows that the co-precipitated protein is mAb414-reactive. **D.** Phosphoimager scan of ^32^P-labeled Immat-C (lane 2) and marker (lane 1) showing that a single protein was labeled. **E.** Phosphoimager scan of precipitations in which ^32^P-labelled Immat-C was incubated with nuclear extracts followed by precipitation of nucleoporins, which were bound to mAb414-coated biomagnetic beads. Capsid-co-precipitation is indicated by the radioactive core proteins on an SDS PAGE. 1: mAb414-coated beads without nuclear extract + capsids 2: Anti capsid coated beads + nuclear extract + capsids, 3: Anti capsid coated beads + nuclear extract without capsids, 4: Uncoated beads + nuclear extract + capsids. The figure shows that Immat-C precipitates Nup153 from the nuclear extract of rat liver.

Figure 1A depicts that importin β became co-precipitated (lane 1). As the nuclear extract did not contain detectable amounts of importin β (not shown) this result implies that the importin β binding activity of the nucleoporin(s) was maintained or restored during extraction and renaturation. Binding was specific as antibody-coated beads without the extracted proteins and uncoated beads failed to interact with importin β. Binding occurred in the absence of an importin β-bound cargo. This finding is in accordance with the observation that importin β after its dissociation from the cargo does not diffuse deeper into the karyoplasm but remains bound to the NPC before being exported into the cytoplasm [40].

The nuclear extract was then subjected to co-immune precipitations using Immat-C which are arrested within the nuclear basket. These capsids contain DNA replication intermediates of the viral genome; they interact with the NPCs [19] and can thus be responsible for nuclear genome amplification. For visualization of co-precipitated proteins we used Sypro Red, staining all proteins after SDS PAGE. As depicted in Figure 1B no co-precipitation could be observed in the absence of nuclear extract. Faint bands were observed in the absence of capsids. This indicates some unspecific binding of nuclear proteins to the carrier beads, probably due to nonspecific hydrophobic interactions. Immat-C-driven pull-down however showed the precipitation of one dominant protein, strongly enriched in comparison to the control extract and the precipitation controls. This protein showed an apparent molecular weight of a ∼180 kDa typical for Nup153 [41],[42]. To confirm the identity of co-precipitated Nup153 we used the antibody mAb414 in Western blot. Figure 1C confirms the Nup153 co-precipitation by Immat-C and provides evidence that neither Nup214 nor Nup358 were co-precipitated.

To further confirm specific Nup153 capsid-interaction we preincubated the nuclear extract with Immat-C prior to precipitation via mAb414 and detection of co-precipitated Immat-C. Since antibodies against denaturated core proteins require very large amounts of protein to provide adequate signals we labeled Immat-C by ^32^P using the protein kinase activity inside the capsids. Phosphoimaging showed that a single protein was labeled having the molecular weight of the core protein of 21.5 kDa (Fig. 1D). As depicted in Figure 1E Immat-C could be precipitated by mAb414-bound Nup. No signal was obtained in the absence of nuclear extract or in the absence of mAb414.

### Interaction between different capsid species and Nup153 domains

**Figure 2:**
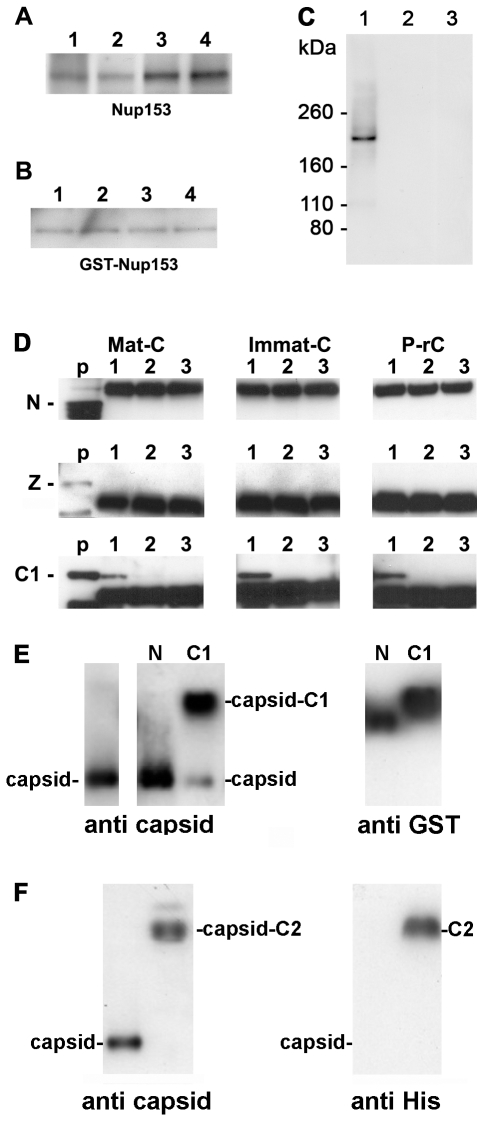
Co-immune precipitations of human Nup153 and Nup153 fragments with different capsids. **A–D.** Capsids were incubated with nuclear extract, GST-Nup153 or Nup153 fragments and precipitated with anti-capsid antibody-coated biomagnetic beads. The panels show immune blots after SDS PAGE. A–C: Detection by mAb414. **A.** 1 Immat-C, 2 Mat-C, 3 P-rC, 4 ΔC-rC. Co-precipitations of Nup153 from nuclear extracts showed that capsids precipitate Nup153 irrespective to the nucleic acid inside the capsid or the presence of the C terminus of the core protein. **B.** Co-precipitations of bacterially expressed GST-Nup153 showed that all capsid species directly interact with Nup153. 1 Immat-C, 2 Mat-C, 3 P-rC, 4 ΔC-rC. **C.** Specificity controls of GST-Nup153 co-precipitation exemplified by P-rC. 1. Positive control. P-rC + GST-Nup153 + anti capsid antibody-coated beads. 2. Without capsids + GST-Nup153 + anti capsid antibody-coated beads. 3. P-rC + without GST-Nup153 + anti capsid antibody-coated beads. **D.** Co-precipitation of GST-Nup153 fragments by P-rC. Immune detection of the Nup153 fragments by anti-GST antibodies bound to anti mouse coated biomagnetic beads. The GST-Nup153 fragment is given on the left of the panels, the capsid type at the top. p: GST-Nup153 fragment without precipitation (positive control). 1: P-rC + Nup153 fragment + anti capsid antibody-coated beads. 2: No capsids + Nup153 fragment + anti capsid antibody-coated beads. 3. P-rC + Nup153 fragment + uncoated beads. The figure shows that only the C1 fragment, which comprises aa 618–999 of Nup153, is precipitated by the capsids. **E.** Gel retardation of P-rC by GST-Nup153 N and C1 fragment on native agarose gels. The migration of GST-Nup153 N and C1 fragments without capsids is shown by anti-GST antibodies (right panel). The migration of the capsid without GST-Nup fragments is shown on the left panel using anti-capsid antibodies. The middle panel shows the capsid migration in the presence of GST-Nup153 N (left lane) and C1 (right lane). **F.** Gel retardation of P-rC by GST-Nup153 C2 fragment. Left: anti-capsid blot, right: anti-His blot. Left lanes: P-rC without Nup153 fragment, right lanes: P-rC with Nup153 fragment.

To determine whether the Nup153 binding was selective for Immat-C we included different capsid species in co-immune precipitations. We used Mat-C, which enter the nucleus and comprise an rcDNA genome; P-rC, which contain *E. coli*-RNA and which is arrested in the basket like Immat-C, and capsids that were formed by C-terminally truncated core proteins (ΔC-rC). ΔC-rC do not contain RNA, cannot be phosphorylated, and cannot enter the basket as these capsids do not contain the NLS. All capsids reacted equally well with the anti-capsid antibody used to precipitate them after preincubation with nuclear extract (not shown). Unexpectedly, all capsids were able to precipitate Nup153 as depicted by immune blot using mAb414 (Fig. 2A).

The Sypro Red stain of co-precipitated proteins was restricted for the molecular weight of the interaction partners. Proteins smaller ∼75 kDa could not be detected as this part of the SDS PAGE was heavily overloaded with high amounts of bead-bound antibodies and BSA used for saturation of unspecific binding sites. We could have thus missed smaller adapter proteins connecting the capsids with Nup153.

To obtain evidence of direct interaction we replaced the nuclear extract by *E. coli*-expressed Nup153 that was purified under native conditions. The fusion protein was previously shown to be functional on nuclear import and export after integration into the NPCs of reconstituted *Xenopus laevis* oocyte nuclei [43]. Figure 2B showed that all capsids precipitated GST-Nup153. As GST does not bind to the capsids (not shown) we conclude that Nup153 interacts directly with the surface of the capsid. The signal was not derived from the capsid preparation since no signal was observed in the absence of GST-Nup153. Furthermore, GST-Nup153 precipitation was not observed in the absence of the capsids implying that GST-Nup153 did not bind directly to the antibody coated-bead (Fig. 2C).

We next set out to determine to which Nup153 domain the capsids bind. We expressed large parts of Nup153 in three fragments (N: aa 53–272; Z: aa 272–543; C1: aa 618–999) as GST fusion proteins. The Nup153 fragments were incubated with the different capsids and co-precipitated by biomagnetic bead-bound anti-capsid antibodies. Co-precipitation of the Nup153 fragments was demonstrated by Western blot using anti-GST antibodies. Figure 2D shows that Mat-C, Immat-C and P-rC failed to precipitate the 50 kDa N terminal part of Nup153. A strong band at 54 kDa was visible in all samples to which the biomagnetic beads were added. This band was most likely the product of an unspecific binding of the secondary blot antibody to the heavy chain of the antibodies used in precipitation. Using the zinc finger domain Z (56 kDa) no precipitation could be observed (Fig. 2D). However all three capsid species co-precipitated the 68 kDa C1 fragment. Binding was specific as no precipitates could be observed in control reactions without capsids or with capsids but without anti-capsid antibodies on the beads. This Nup153 fragment comprises an importin β-binding domain [44],[45] thus implying that importin β and capsids compete for Nup153 binding.

A fourth His-tagged fragment (C2: aa 992–1219) containing most of the ∼30 FXFG repeats [46] was analyzed by co-precipitation but we observed an unspecific binding to the beads. To circumvent this technical problem we performed retardation gels. We incubated P-rC with His-Nup153 C2 and separated the complex by native agarose gel electrophoresis. The Nup fragment was visualized by anti-His antibodies, the capsid by anti-capsid antibodies. Control experiments were performed using the N and the C1 fragment. Migration of the fragments was determined by anti-GST antibodies.

Figure 2E confirmed that the N fragment did not interact with the P-rC as no shift of capsid migration could be observed upon the presence of this Nup153 domain. Nup153 C1 caused a retarded migration of the majority of capsids (Fig. 2F) and fragment C2 retarded the migration of all P-rC (Fig. 2F). Although fragment C2 is largely hydrophobic we assume that the capsid binding is not unspecific as the capsids are negatively charged and migrate on native agarose gels as ∼3000 bp linear double stranded DNA fragments.

As both fragments C1 and C2 barely overlap we conclude that there is more than one interaction site on Nup153.

### Competition of P-rC, Nup153 and importin β

**Figure 3:**
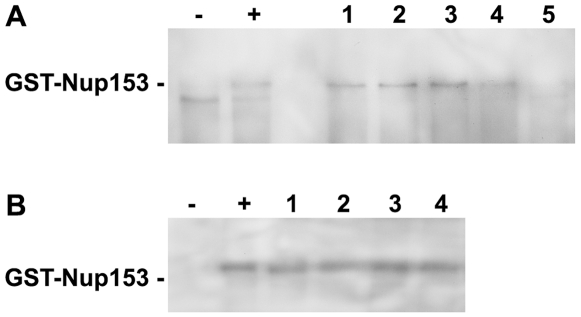
Competition of P-rC, Imp β and Nup153 interactions. **A.** Nup153 was preincubated with importin β and P-rC. The complex was precipitated by anti capsid antibody-coated beads and Nup153 was detected by Immune blot using mAb414. − negative control without P-rC, +: positive control in the absence of Imp β. 1–5. Different ratios of capsid-importin β. 1: 1∶33, 2: 1∶50, 3: 1∶100, 4: 1∶150, 5: 1∶300. **B.** Anti capsid antibody-coated beads were preloaded with P-rC. P-rC was then saturated with Nup153 followed by addition of different excesses of Imp β.−: and +: as in A. Molar ratio capsid-Imp β 1: 1∶2600, 2: 1∶4000, 3: 1∶5400, 4: 1∶6700. The figure shows that even an excess of Imp β cannot displace Nup153-bound capsids. After Nup153 attachment to the capsid even excessive amounts of Imp β cannot replace Nup153.

*In vivo*, numerous proteins and nuclear factors pass the NPC simultaneously requiring interaction with Nup153. As Immat-C and P-rC are arrested *in vivo*, one should expect that the affinity of the physiological transport complexes to Nup153 is weaker than that of the capsids. We incubated capsids (P-rC) immobilized on biomagnetic beads with Nup153 and importin β in different ratios. Figure 3A, lane 1, shows that no GST-Nup153 bound to the beads in the absence of capsids thus demonstrating the specificity of Nup153 binding. When incubating Nup153 in parallel to different amounts of importin β with the capsids only molar importin β excesses of more than 150-fold with regard to Nup153 prevented Nup153 binding to the capsids. This result thus confirm that capsid and importin β binding sites on Nup153 overlap and show further that the capsid binding was much stronger than the importin β interaction.

We next asked whether bound capsids can be displaced from Nup153 by importin β. We preincubated biomagnetic bead-bound P-rC with Nup153 and added importin β after removal of unbound Nup153. Figure 3B shows that even 6700-fold molar excesses of importin β did not remove Nup153 from the capsid.

### Silencing of Nup153 expression by siRNA

**Figure 4:**
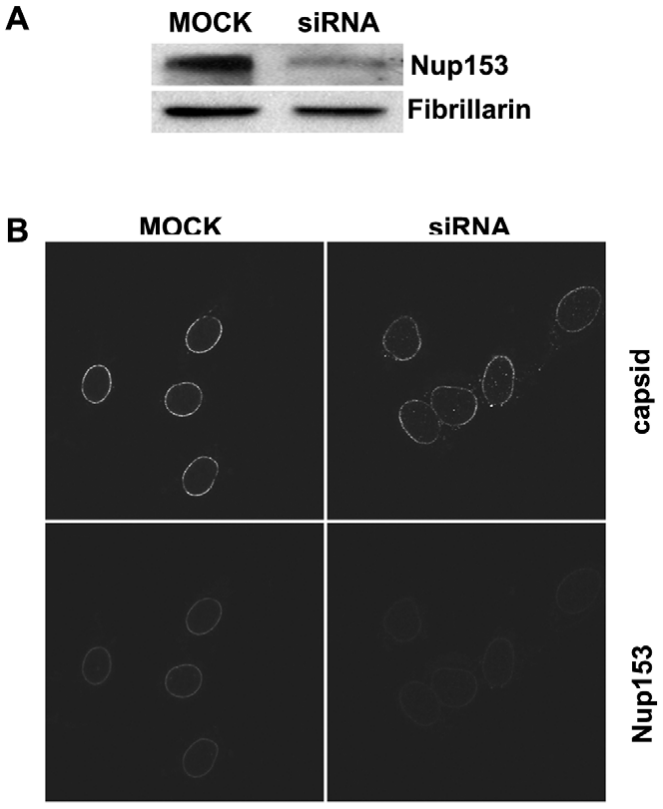
Nuclear import of P-rC in nuclei of partially Nup153-silenced cells. **A.** Immune blot of Nup153 in Nup153-silenced cells. Upper panel Nup153, lower panel: the house keeping gene Fibrillarin. The blot shows a significantly reduced Nup153 expression **B.** Nuclear import in digitonin-permeabilized cells. Left: control mock-transfected cells. Right: Nup153-silenced cells. Capsids and Nup153 are visualized by indirect immune fluorescence (see as well video in *Supporting information*). Consistent with the incomplete Nup153 knock down some P-rC entered the nucleus exclusively in Nup153 siRNA-treated cells.

In order to determine whether the capsid arrest in the basket is mediated by Nup153 we suppressed Nup153 expression in HeLa cells by siRNA. We controlled Nup153 expression in parallel to the expression of Fibrillarin, which is a component of a nucleolar small nuclear ribonucleoprotein (SnRNP) involved in ‘*house keeping*’ for nucleolar integrity [47]. Figure 4A shows that Fibrillarin expression was in fact unaltered but Nup153 reduced by 80%. Suppression was limited because Nup153 is essential for cell viability.

After permeabilization of these cells by digitonin a nuclear import assay using P-rC was performed. We visualized Nup153 and capsids using indirect immune fluorescence. Nup153 staining was weaker in 80–90% of the siRNA-treated cells than in non-silenced cells (Fig. 4B, lower panel). The nuclei of these cells were larger than in mock-transfected cells most probably due to the role of Nup153 in mitotic progression [48]. The nucleoporin Nup153 plays separate roles in both early mitotic progression and the resolution of mitosis.

In the nuclei of the control cells, P-rC accumulated at the nuclear envelope and did not enter the karyoplasm as demonstrated previously [18]. In contrast, a proportion of capsids were found in the karyoplasm of Nup153 siRNA-transfected cells, (still image: Fig. 4B upper panel; for videos see Videos S1, S2, S3 and S4). Quantification on 20 cells revealed that 18–19% of capsids entered the nucleus while ca. 80% were still arrested at the NE.

### Effect of cross-linking on nuclear import of Mat-C

**Figure 5:**
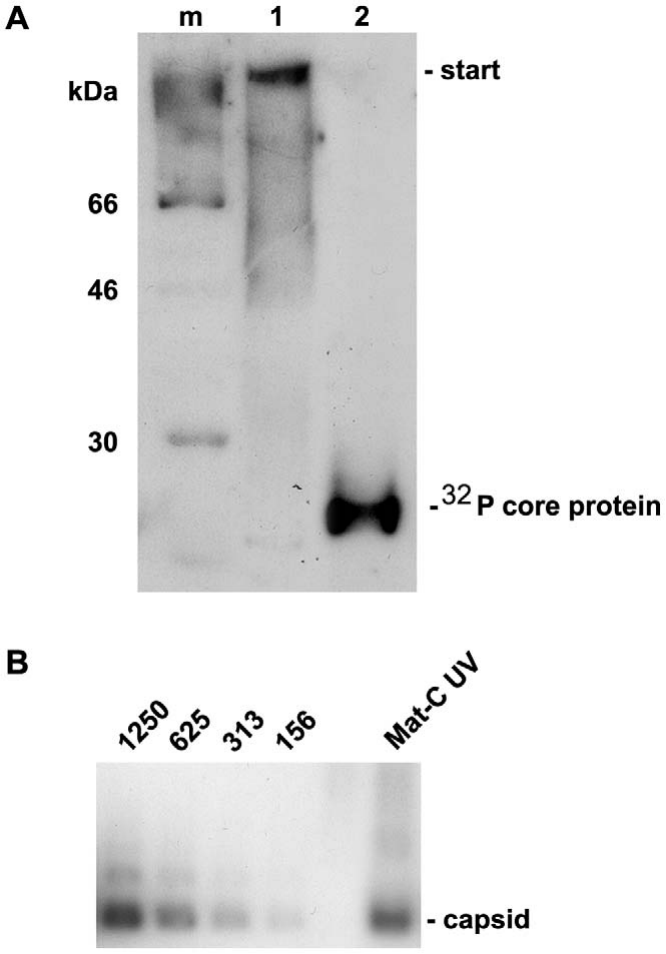
Analysis of cross-linked core particles. **A.** Lane 1: ^32^P-labelled Mat-C-UV and (lane 2) ^32^P-labelled Mat-C were separated on a 4–12% SDS-PAGE. Phosphoimaging showed that the core proteins of Mat-C-UV did not enter the separating gel indicating successful cross-linking. In contrast the core proteins of Mat-C migrated as a 21.5 kDa band. m: ^14^C-labelled molecular weight marker. **B.** Immune blot of Mat-C-UV and non cross-linked P-rC after native agarose gel electrophoresis. Mat-C-UV migrated as the P-rC standard indicating that the core proteins were linked within the individual capsids and that the capsids were not linked to each other. The identical migration indicates that UV irradiation has not changed the surface charge. The numbers on top of the standard dilution series give the amount of the P-rC in pg.

We next asked how Mat-C proteins could enter the nucleus despite the strong binding to Nup153. We followed the hypothesis that capsids should remain intact while genome maturation continues but should liberate the genome from the basket once rcDNA is formed. To test this hypothesis we cross-linked the Mat-C subunits, which were ^32^P-labelled by UV irradiation (Mat-C UV) and analyzed their integrity. Figure 5A shows that the cross-linking caused a strong reduced migration of the core protein subunits in SDS PAGE. Most of the protein was retained at the entry site of the SDS PAGE; only traces resulted in a smear >45 kDa. To test whether cross-linking occurred between capsids, which would have caused unsuitable particle aggregates we separated Mat-C UV on native agarose gel and detected them by anti-capsid antibody used before. We observed no difference in migration compared to the P-rC standard (Fig. 5B), demonstrating that UV irradiation only induced bonds between subunits of individual capsids. The result further shows that the UV treatment had not changed the surface charge.

We next investigated the transport competence of Mat-C UV in comparison to untreated Mat-C. We injected 1×10^7^ capsids into the cytosolic periphery of 6 *Xenopus laevis* oocytes and followed the fate of the capsids by electron microscopy. We used *Xenopus laevis* oocytes sine more NPCs can be analyzed by EM in this system than by using permeabilized cell lines thus giving more reliable results. Combining these two systems and comparing the results is however justified since, as far as is known, nuclear import is identical in both systems [49],[50].

**Figure 6:**
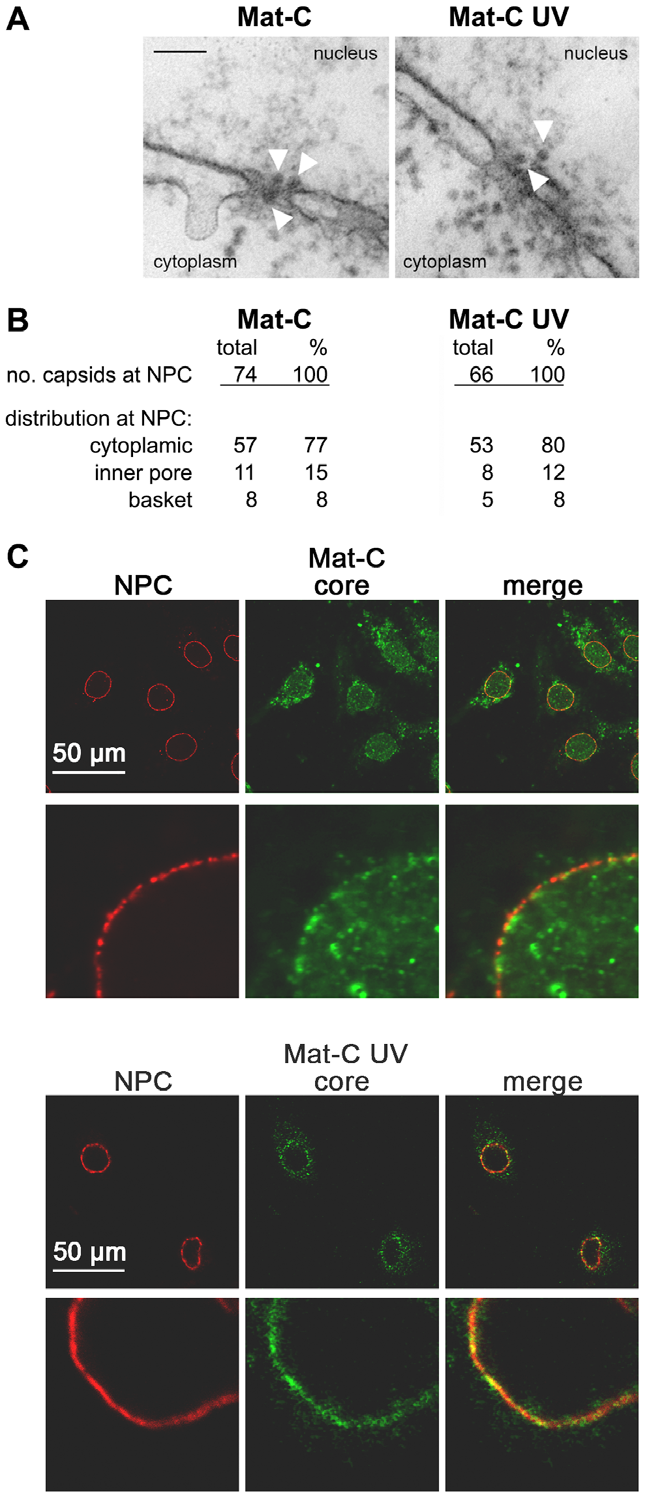
Nuclear transport of Mat-C and Mat-C-UV. **A.** Electron microscopy of the capsids at the NPCs after microinjection into the cytoplasm of *Xenopus laevis* oocytes. The white arrows indicate capsids. Black scale bar: 100 nm. **B.** Frequency of NPC-attached capsids and the capsid distribution at the NPCs. Both capsid species showed a similar frequency at the NPCs and similar importation into the nuclear basket. **C.** *In vitro* transport assays of the capsids in digitonin-permeabilized HuH-7 cells. NPCs (red) and capsids (green) are visualized by indirect immune stain. The merges are depicted on the right panels. The panels show overviews and strongly magnified images. While Mat-C (upper two panels) caused intranuclear fluorescence cross-linked capsids (lower two panels) failed to enter the karyoplasm.

As control, six *Xenopus laevis* oocytes were injected with Mat-C. We restricted the incubation time after injection to 1 h, the earliest time point at which significant numbers of capsids arrive at the nuclear pore (not shown). Such a short time was chosen in order to detect possible differences in nuclear entry of the capsids. As shown in Figure 6A both types of capsids entered the nuclear basket as intact particles. We determined the number of capsids that arrived at 68 NPCs (Mat-C) and 74 NPCs (Mat-C UV), and determined their location at the NPCs. Figure 6B showed that a similar number of capsids arrived at the nuclear pore indicating that the cross-link neither affected the intracytoplasmic transport capacity nor the interaction with the NPCs. We next analyzed the distribution of the capsids, showing that both capsid species exhibited the same distribution at the NPCs with a majority on the cytoplasmic face. To our experience this dominantly cytoplasmic localization is related to the short incubation time. However, the same proportion of Mat-C and Mat-C UV entered the pore and were found in the nuclear basket, implying that their transport competence was the same.

We next analyzed whether Mat-C UV diffuses deeper into the nucleus like untreated Mat-C or remained arrested at the nuclear envelope like Immat-C and P-rC. These assays were performed with digitonin-permeabilized cells since the relatively low amounts of capsids would have been undetectable in *Xenopus laevis* oocytes. Capsids were detected by indirect immune fluorescence using the same anti-capsid antibody that showed an unchanged reactivity after cross link. Figure 6C shows that Mat-C entered the nucleus as it was previously reported for different permeabilized cells and in living cells [19],[23]. In contrast Mat-C UV failed to enter the karyoplasm and remained associated at the NPCs and some cytosolic structure. We assume that the cytosolic binding sites are collapsed microtubules as such binding was demonstrated before for digitonin-permeabilized cells [23].

### Effect of Nup153 – capsid interaction on nuclear import of other cargos

Figures 2 and 3 showed that the capsids bound to a Nup153 domain that participates in importin β binding and that the interaction competes with importin β *in vitro*. To obtain *in situ* evidence on Nup153 that is integrated into the NPCs we analyzed whether binding of capsids to Nup153 interfere with the importin β-dependent nuclear import pathway. These experiments were performed with P-rC because the large numbers of capsids required for Nup153 saturation on the permeabilized cells were only available for this type of capsid. We used the human hepatoma cell line HuH-7 cells which is able to synthesize hepatitis B virions after transfection with HBV DNA.

We incubated digitonin-permeabilized cells with different amounts of P-rC. Nuclear import into the basket was facilitated by means of the nuclear transport receptors in rabbit reticulocyte lysate. After incubation cells were washed. In the last washing buffer no P-rC was detectable.

First we quantified the amount of NPCs and the number of bound capsids. NPC number was calculated from the Nup153 signal obtained by Western blot, which was compared to a dilution series of *E. coli*-expressed Nup153 (not shown). We determined ∼6000 NPCs per HuH-7 nucleus, which is higher than the ∼2770 NPCs found in HeLa cells [9]. It is however known that the NPC number varies strongly between different cell types (18,451+/−2,336 (Purkinje cells) to 402+/−67 (oligodendrocytes) [51].

We used P-rC preloaded digitonin-permeabilized cells after washing and added new cytosol together with two fluorescent cargos – Alexa594 NLS-BSA and Alexa647 M9-BSA. NLS-BSA is imported by importin β while M9-BSA uses transportin for nuclear entry. Both cargos comprise the same number of nuclear localization signals, as determined previously [18]. After import reaction, capsids and NPCs were stained by indirect immune fluorescence, and nuclear cargo concentrations were determined semi-quantitatively using confocal laser scan microscopy. Quantification was performed in the equatorial region of the nuclei. A positive control was performed on cells that were preincubated in the absence of capsids but to which the cargos were added in a second step and incubated at 37°C. In the negative control no P-rC was added during the first incubation but the following import reaction with the fluorescent cargos was performed on ice, thus inhibiting active nuclear transport [52].

**Figure 7:**
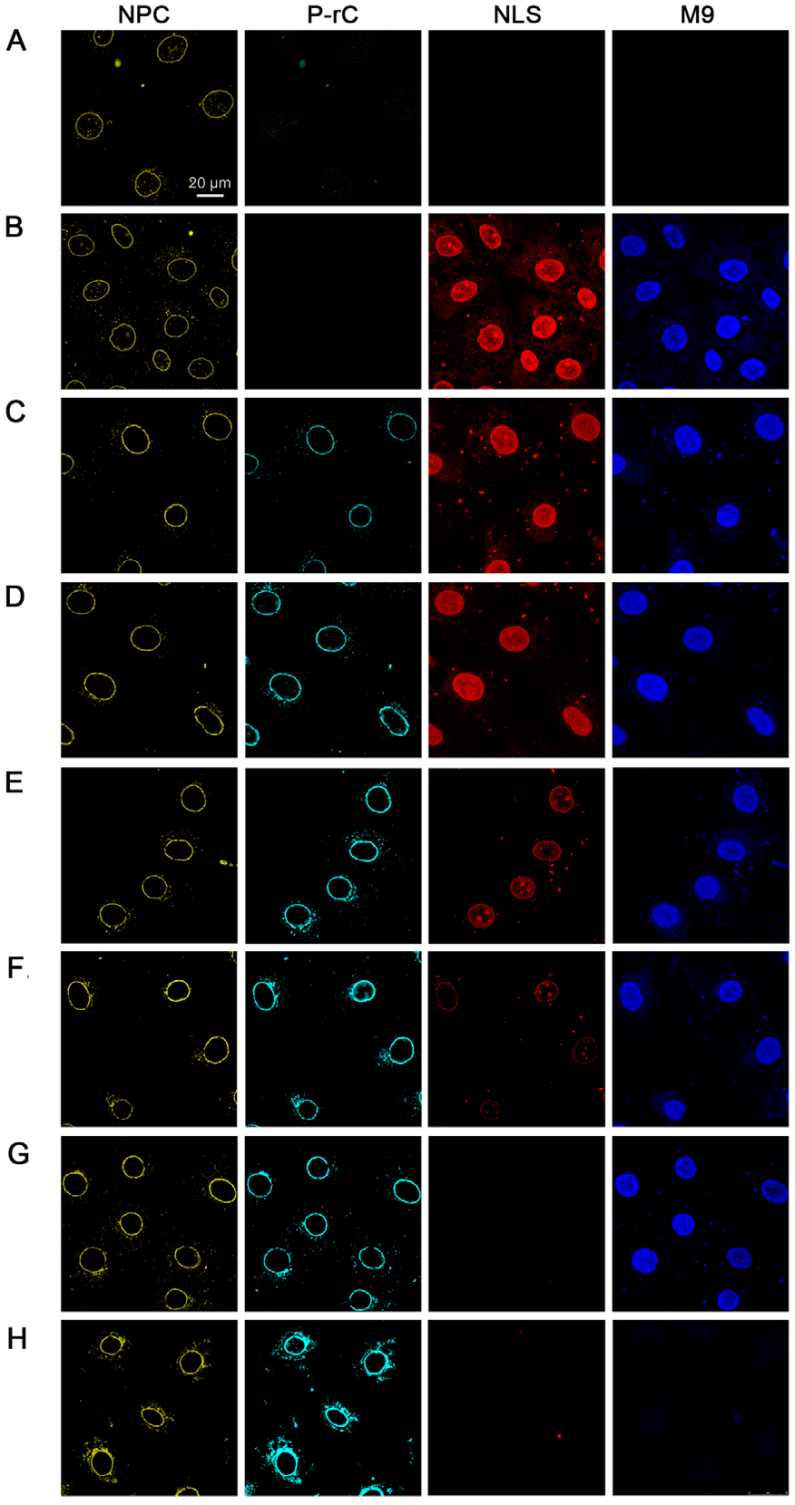
*In vitro* transport assays of NLS- and M9-linked fluorescent BSA conjugates after preload of the NPCs by P-rC. NPCs (yellow) were visualized by indirect immune fluorescence using mAb414. P-rC (cyan): Indirect capsid immune fluorescence using anti capsid antibodies. The panel show increasing signal strength related to the amounts of capsids subjected to the cells. NLS (red): fluorescence of Alexa594 NLS-BSA. M9 (blue): Fluorescence of Alexa647 M9-BSA. While the NLS-import rapidly decreased with increasing preload of the NPCs with P-rC, the M9-import was less affected. **A.** Negative control at 4°C without capsid preload. **B.** Positive control at 37°C without capsid preload. **C.** Preload of the nuclei with 25 ng P-rC, **D.** 100 ng P-rC, **E.** 400 ng P-rC, **F.** 1600 ng P-rC, **G.** 6400 ng P-rC, **H.** 12800 ng P-rC. All pictures were taken at the same magnification. Space bar: 20 µm.

Figure 7 shows no intranuclear fluorescence in the negative control (Fig. 7A) but strong signals for both cargos in the positive control (Fig. 7B). With increasing preload of the nuclei by capsids the import of both cargos became reduced (Fig. 7C–H). At the highest capsid concentration no intranuclear fluorescence could be observed (Fig. 7H). In this sample the average nuclear diameter increased from 200 µm^2^ in the negative control to 240 µm^2^. This observation is in accordance with a “plugging” of the NPCs by the capsids, which does not allow exchange of smaller molecules needed for equilibration of the osmotic pressure between nucleus and reaction mixture.

Figures 7B to G show further that the reduction of nuclear import appeared for NLS-BSA at lower preloaded numbers of capsids than for M9-BSA. As no unbound P-rC was present after washing, we can exclude competition between capsid NLS and NLS-BSA for the transport receptors importin α and β.

**Figure 8:**
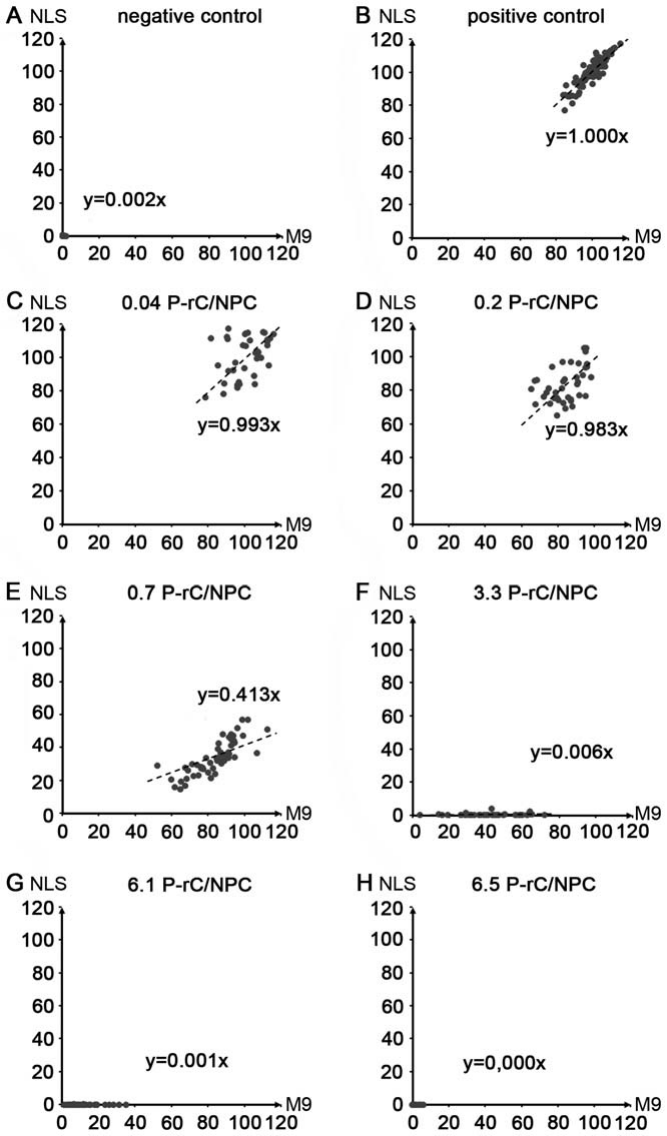
Scatter plots of the relative intranuclear concentrations of M9- and NLS-BSA in individual cells preloaded with P-rC. Each point represents one cell. X-axis: Intranuclear concentration of the M9-BSA. Y-axis: Intranuclear concentration of NLS-BSA. Both axes give the relative intranuclear cargo concentration in %. The dotted lines show the regression lines. The corresponding slope is given in each panel. The average number of capsids per NPC is given on top of each panel. **A.** Negative control at 4°C. **B.** Positive control without capsid preload. **C.–H.** Increasing amounts of P-rC as in Figure 8. 100%: mean value of the positive import reaction. Only those cells were analyzed in which the section was within the equatorial region (570 cells total).

We next quantified the import in 570 nuclei. The mean intranuclear fluorescence in the positive control was taken as 100%. Figure 8 shows scatter plots obtained for each capsid preload. We observed a significant variation between the nuclei in one sample, for instance between 80% and 120% in the positive control. This can be explained by variable nuclear permeability throughout the cell cycle [53]. Moreover, this observation confirms that the nuclei remained intact during the transport reaction as a disruption would have resulted in equal concentrations. The import via transportin and via importin β was correlated in all cells and followed a Gaussian normal distribution. As indicated by the slopes of the regression lines no significant changes were observed at preloads from 0 to 0.2 capsids per NPC (Fig. 8B–D). With larger numbers of capsids, the importin-mediated import became greatly reduced (Fig. 8E; 0.7 P-rC/NPC) or undetectable (Fig. 8F; 3.3 P-rC/NPC), while the transportin-mediated pathway remained significantly more active (p≤10^−9^). Concentrations ≥3.3 capsids/NPC blocked both the NLS-and M9-mediated import, most likely the result of steric hindrance by capsids that got stuck in the channels of the NPCs. Collectively the data however indicate that blocking the capsid binding sites on Nup153 interferes with the importin β-mediated nuclear entry but not with the transportin pathway.

## Discussion

Karyophilic cargos interact transiently with components of the NPC via transport receptors before they are released to the karyoplasm. HBV capsids are an exception as they remain arrested in the nuclear basket. We showed that HBV capsids bound solubilized and renatured rat Nup153 from a nuclear extract with importin binding activity. This is consistent with the high degree of conservation of Nups among different species and the conserved mechanisms of nuclear translocation [54]. It confirms further the observation that Immat-C is arrested at the NPCs of different cell lines [19].

Neither co-purification of Nup98 or Nup160, which connect Nup153 with the central frame work of the NPC [55] nor of Tpr (268 kDa), which is attached to the NPC via Nup153 [7], was observed. This finding indicates that the NPC components became disassembled during purification and did not reassemble upon renaturation. This interpretation is in accordance with previous findings that NPC reconstitution depends on the presence of RanGTP [56]. RanGTP has a low molecular weight of 25 kDa and is removed from the nuclei prior to disintegration of the NPCs. The absence of a 97 kDa band in the co-precipitation further confirms that the binding was not mediated by importin β, which is in accordance with the direct binding observed in co-precipitation of natively purified GST-Nup153. The latter finding provides further evidence that the binding did not require a protein linker, which may have been below the 75 kDa limit of the Sypro Red stained gel.

HBV capsids undergo complex modifications upon genome maturation which are not well understood. Their changes comprise not only the reverse transcription of the encapsidated RNA to rcDNA but also protein phosphorylation [31],[32] and eventually dephosphorylation. We found that Nup153 was precipitated by Immat-C, containing replication intermediates and possibly empty capsids, and by Mat-C containing rcDNA. This observation suggests that the type of nucleic acid within the capsid has no impact on Nup153 interaction. The observation that *E. coli*-expressed RNA containing capsids that were phosphorylated *in vitro* by protein kinase C and empty *E. coli*-expressed capsids that lacked the RNA-binding and phosphorylated C-terminal domain interacted equally with Nup153 shows that the binding is mediated by the N-terminal assembly domain. It further confirms that importin β, which interacts via importin α with the NLS on the C-terminal domain [18] do not interfer with capsid binding to Nup153.

Searching for the domain on Nup153, which interacts with the capsids we observed that aa 53–543 of Nup153 did not show any interaction. This part of Nup153 comprises a transportin binding site (aa 247–290 [57]). In contrast aa 618–999 and 992–1219 – both comprising FxFG repeats - interacted with the capsids. As other FxFG repeat comprising Nups were not co-precipitated we conclude that the binding is specific for Nup153 and not based on hydrophobic interactions.

To obtain evidence of overlap between importin β and capsid binding sites we performed competition experiments in the presence of excesses of importin β. The strong binding with regard to importin β is consistent with an attachment of the capsids to the FXFG repeats as these sites interact with this import receptor. This strong affinity could explain the mechanism by which the capsids can remain arrested on Nup153 within the cells despite of the 1000 exchange reactions that pass each nuclear pore per second [4].

Partial silencing Nup153 showed that a significant proportion of P-rC entered the nucleus. The incomplete entry is consistent with the high affinity of the capsids to Nup153, assuming that less than 8 copies of Nup153 are sufficient for capsid arrest. Considering that Nup153-linked Tpr was not precipitated the result thus confirms that no other nucleoporin has a significant effect on the abortion of the capsid entry into the nucleus.

Our results showed additionally that the structural part of the capsid caused the interaction, which has a well ordered structure. Within 16 Å resolution no differences could be found between *E. coli*-expressed capsids, Mat-C and Immat-C [28] although one group reported a hydrophobic pocket on the capsid surface that exists on Mat-C only [58]. We considered structural causes to be an unlikely explanation for the different entry behavior and followed the hypothesis that Immat-C is more stable than Mat-C. Consequently, we assumed that maturation-dependent disintegration of Mat-C leads to capsid subunits which may diffuse from the NPC to the karyoplasm. It was recently shown that capsids may disintegrate to core protein dimers [59]. Irrespective of the T = 3 or T = 4 symmetry of the capsids, the core protein dimers would exceed the number of Nup153 molecules per NPC by a factor of >10. Supernumerous subunits could diffuse into the karyoplasm where they reassemble [59]. Our nuclear import assays with cross linked capsids confirmed this hypothesis showing that disassembly is a prerequisite for intranuclear capsid formation. These data are also consistent with recent observations that nuclear entry of capsid and genome is independent upon RanGTP [19], as a dissociation of the import receptors is not required.

In order to analyze the functional effects of capsid binding to Nup153 *in situ* we measured nuclear import via the importin β pathway. Our observation that the NLS-BSA import was impaired to a much greater degree than M9-BSA translocation provides evidence for the concept that transport inhibition was specific by blocking importin β binding sites and was not the result of steric interference. The strong inhibition of the importin β pathway is in accordance with observations of Walther et al. [43] who reported that Nup153 is essential for nuclear entry via importin β but not via transportin. In fact our approach of inhibiting Nup153 function with capsids allows for the first time the confirmation of these data without interfering with the NPC architecture, i.e. Nup50 translocation or Tpr depletion [5],[7].

**Figure 9:**
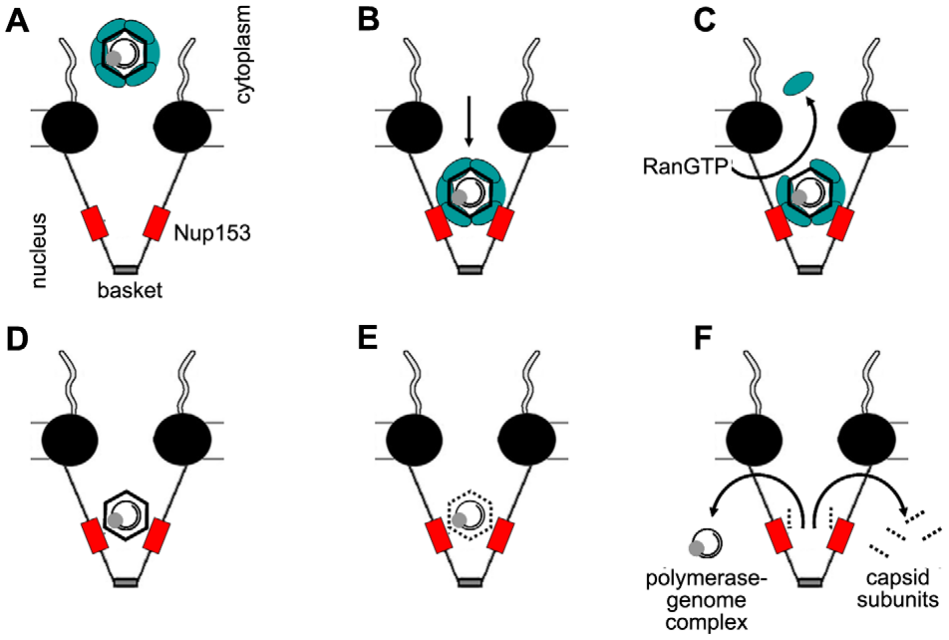
Schematic model of intranuclear basket events leading to genome release from the hepatitis B virus capsid. **A.** Capsids in complex with the nuclear transport receptors Import α and β attach to the nuclear pore. **B.** The complex passes the nuclear pore and becomes arrested by interaction between importin β and Nup153. **C.** RanGTP dissociates the nuclear transport receptors from the capsid and recycles them into the cytoplasm. **D.** After removal of the nuclear transport receptors Nup153 interact directly with the capsid. Immature Capsids remain in the arrested statebut eventually undergo further maturation. **E.** Mature capsids disintegrate. **F.** Capsid subunits, which are supernumerous to the Nup153 copies, diffuse into the nucleus. The polymerase-viral DNA complex leaves the basket. The central framework of the NPC is shown as black spheres, Nup153 as red boxes, Import α and β complex as green ellipse. The capsids are depicted as hexagons, containing the rcDNA genome (black circles) and the polymerase (grey sphere).

Based on our observations we propose a model of nuclear entry for the HBV genome, which strikingly differs from that for any other virus. According to previous data we postulate that the viral genome is transported within the capsid via microtubules to the nuclear periphery [23]. This assumption does not exclude that the capsids undergo a limited disintegration and re-association of some capsid subunits. Such capsid “breathing” is in accordance with *in vitro* findings [60],[61] and can explain the observation of polymerase-free hepadnaviral DNA genomes [62], which are nonetheless precipitable by anti capsid antibodies [63]. After binding to importin α/β the capsids are transported into the nuclear basket (Fig. 9). This transport requires the DNA minus strand synthesis which is linked to NLS exposure by some core subunits [19]. The import complex binds to Nup153 via importin β, followed by importin α and importin β dissociation, which is mediated by RanGTP. Due to their affinity to Nup153 capsids interact directly with Nup153. While Mat-C disintegrates, Immat-C remain in the arrested state. *In vivo* these arrested capsids should not interfere with the viability of the hepatocytes [64] as thousands of capsids would be necessary to significantly block the nuclear pores. In fact, only high level expression of capsids in cell lines causes toxic effects [65]. It is plausible to assume that genome maturation can proceed in Immat-C but experimental evidence is difficult to obtain as genome maturation is slow. At the beginning of infection, when significant amounts of surface proteins have not yet been synthesized, ongoing genome maturation after arrest prevents premature release of the genome into the nucleus. Due to the dependence of the second strand DNA synthesis on the environment within the capsids [66], premature release of minus strand DNA would lead to a loss of viable virus. Thus, the maturation dependent arrest of HBV capsids is probably an essential step in the life cycle of the virus.

## Materials and Methods

### Preparation of nuclear proteins

Nuclear proteins were prepared from rat liver nuclei using urea [67] followed by protein refolding during dialysis. Eight gram of rat liver were minced in 16 ml of ice-cold 250 mM sucrose in TKM buffer (50 mM Tris-HCl, pH 7.5, 25 mM KCl, 5 mM MgCl_2_, protease inhibitor mix (complete) (Roche)). All subsequent preparations were performed on ice. The tissue was homogenized using a motor-driven Teflon pestle with 10 strokes at 1700 rpm. The homogenate was filtered through two layers of a filter (59 µm mesh). Three ml of homogenate were mixed with 6 ml 2.3 M sucrose/TKM buffer and loaded on a 1 ml cushion of 2.3 M sucrose/TKM buffer. After centrifugation for 30 minutes at 124,000×g at 4°C, the pellet was resuspended in 200 µl TKM buffer. The resuspended nuclei were treated with 4 ml 8 M urea for 1 h at 65°C and subjected to dialysis at 4°C against 1.5 l 1×PBS/2 mM DTT) for 48 h. During the dialysis the buffer was changed four times. The protein concentration was determined by bicinchonic acid (BCA) assay. The nuclear proteins were frozen in liquid nitrogen and stored in aliquots at −80°C. This lysate did not contain detectable amounts of importin β, presumably excluding interference of importin β with capsid binding (not shown).

### Expression and purification of GST-Nup153 and Nup153 fragments

GST-Nup153 was expressed using the vector pGEX 2T-hNup153, which encodes for human wt Nup153 [43]. Expression was performed in *E. coli* BL21 CodonPlus RIPL (STRATAGENE). Bacteria were grown at 37°C in 2×YT-medium/100 µg/ml ampicillin/30 µg/ml chloramphenicol/ 0.2% glucose to an OD_600_ of 1.0 and cooled to 18°C prior to induction of the expression by 1 mM IPTG and incubation for 16 h at 18°C. PMSF was added to 2 mM and the bacteria were chilled for 15 min at 4°C followed by sedimentation at 4000 g for 15 min at 4°C. The pellet was resuspended in PBS/1 mM DTT/1×EDTA-free complete protease inhibitors (Roche Applied science). Bacteria were lysed by sonification on ice. The lysate was cleared 30 min 15000×g at 4°C. One milliliter equilibrated GST-Sepharose High Performance (GE-Healthcare) was added to the supernatant and incubated for 1 h at RT followed by o.n. incubation at 4°C. The solution was transferred to a polypropylene column (BIO-RAD). The matrix containing the GST-Nup153 was washed with 30 ml binding-/washing buffer. GST-Nup153 was eluted using 5 ml 20 mM Glutathione/200 mM NaCl/50 mM Tris (pH 7.5)/1 mM DTT/1×EDTA-free complete protease inhibitors. The eluate was dialyzed against 200 mM NaCl/50 mM Tris-HCl pH 7.5/50% Glycerol/250 mM Sucrose/1 mM DTT for 24 h at 4°C. The buffer was changed three times. GST-Nup153 was stored in aliquots at −80°C.

The N terminal human Nup153 fragment was expressed using the vector pGEX-153N (53–272), the zinc finger domain using the vector pGEX-153Z (272–543) and the N terminal half of the C terminus using the vector pGEX-153C1 (618–999). Expression was performed using *E. coli* BL21 (DE3)Lys using the protocol of [68] Bacteria were grown in NZy (rich) medium and expression was performed for 3 h at 37°C. The sedimented bacteria were lysed as described above. Purification was performed as described above.

The C terminal half of the C terminus (aa 992–1219) was expressed as a His-tagged protein using the vector pET28-153C2 [68]. The His-tagged fragment was expressed by using *E. coli* BL21 (DE3)Lys in LB/Kanamycin medium. Bacteria were lysed as described above. The precleared lysate was purified by Ni^++^ agarose (Quiagen) using the protocol of the vendor. After elusion, the His-tagged Nup fragment was renatured by dialysis (1 h against 1 M urea, 0.2 M NaCl, 0.1 M NaH_2_PO_4_ pH 7.6, 0.5 mM PMSF, 1 h dialysis against 0.2 M urea, 0.2 M NaCl, 0.1 M NaH_2_PO_4_ pH 7.6, 0.5 mM PMSF, 1 h dialysis against 0.2 M NaCl, 0.1 M NaH_2_PO_4_ pH 7.6, 0.5 mM PMSF and 1 h against 0.2 M NaCl, 0.1 M NaH_2_PO_4_ pH 7.6).

### Preparation of capsids

P-rC was generated by *in vitro* phosphorylation of *E. coli*-derived capsids using protein kinase C and [γ^32^P] ATP to determine the success of the reaction [69]. *E. coli*-derived capsids were expressed and prepared as described previously [20]. Quantification of capsids was done by immune blotting of the capsids after native agarose gel electrophoresis [18]. Preparation of Mat-C from virions from the permanently virion-expressing hepatoma cell line HepG2.2.15 [70] was done according to Rabe et al. [19]. This cell line expresses infectious HBV [70]. Immat-C was purified from the same cell line using the following protocol. Ten 16 cm dishes of HepG2.2.15 cells were grown in DMEM medium, containing 10% FCS until 80% confluence. The medium was replaced by DMEM/1% FCS and cells were grown for an additional 4 days. Cells were washed twice in PBS, harvested by a rubber policeman and sedimented for 5 min at 200×g and 4°C. Cells were resuspended in 0.1% Nonidet P-40/PBS and sonified on ice. Cellular nucleic acids were digested by addition of 20 U/ml DNase I/20 µg/ml RNase A in 15 mM MgCl2 for 15 min at 37°C. This short incubation was chosen as capsid instability may allow entry of nucleases upon longer incubation periods [60],[61]. The lysate was centrifuged for 20 min at 10,000×g. The supernatant was adjusted to 0.75% Nonidet P-40/5 mM CaCl_2_/20 U/ml S7-Nuklease and incubated for further 15 min at 37°C before EDTA was added to 15 mM. The lysate was centrifuged for 10 min at 4°C and 18,000 g. The capsids in the supernatant were loaded on a 1 ml 25% (w/v) sucrose cushion and sedimented for 2 h at 10°C and 50,000 rpm in an SW60 rotor (Beckman). The sediment was resuspended in 500 µl PBS, centrifuged 5 min at 4°C and 12,500×g. The capsid-containing supernatant was adjusted to 2 mM DTT in order to prevent disulfide bond formation and stored in aliquots at −80°C.

### UV-cross linking of mature HBV capsids (Mat-C UV)

Mat-C were labeled by [γ^32^P] ATP as described elsewhere [69]. Labeled capsids were exposed 6×8 min to 254 nm UV light on ice (Stratagene UV Stratalinker; 1 cm distance to the UV source, ∼4000 µW/cm^2^). Samples withdrawn before and after cross-linking were analyzed by SDS-PAGE with a subsequent exposure to a phosphoimaging screen. Evaluation of a possible inter-capsid cross-link was done by native agarose gel electrophoresis and subsequent immune detection [18].

### Immune precipitation and detection of co-precipitated proteins

For immune precipitation 1.2×10^7^ anti-rabbit antibody-coated biomagnetic beads (Invitrogen) were added to 185 µg anti-HBV capsid antibody (DAKO). The volume was adjusted to 1000 µl by addition of 0.1% BSA/PBS and incubated overnight at 4°C on a rotating wheel. Unbound antibodies were removed by washing the beads three times in 0.1% BSA/PBS. For inverse immune precipitation, 12.5 µg mouse monoclonal antibody 414 (mAb414, HISS) were bound to 1.2×10^7^ anti-mouse antibody-coated biomagnetic beads as described for the anti-capsid antibody.

For co-immune precipitations of the nucleoporins 100 ng of HBV capsids were preincubated with 75 µg nuclear proteins for 2 h at 37°C in transport buffer (20 mM Hepes, pH 7.3, 2 mM MgAc, 110 mM KAc, 5 mM NaAc, 1 mM EGTA, 2 mM DTT, protease inhibitor mix (complete) (Roche)) in a final volume of 250 µl. The precipitation mixture was incubated with constant shaking at 37°C overnight. Afterwards the beads were washed three times in 500 µl 0.1% BSA/PBS, 1×500 µl 0.1% Nonidet P-40/PBS, and transferred into a new cup. After three additional washing steps with 500 µl PBS, the pellet was resuspended in 20 µl 1× loading buffer (Anamed), denatured for 10 min according to the vendor and loaded on the SDS gels. (Invitrogen and Anamed). For detection of total proteins that were co-precipitated Sypro Red staining was performed according the manufacturer.

For immune detection the proteins on the gel were transferred to a PVDF membrane (VWR International) by electroblotting. The membrane was blocked for 1 h at RT in 5% fat-free milk powder in PBS. For detection of co-precipitated nucleoporins the first antibody (mAb414, HISS) was added at a dilution of 1∶3000 in 5% fat-free milk/PBS for 3 h at RT. After washing three times in 0.1% Tween-20/0.5% milk/PBS the membrane was incubated for 1 h with a horse radish peroxidase anti-mouse antibody (0.16 µg/ml; Dianova). Detection was performed by ECL (PerkinElmer).

### Co-immune precipitations of Nup153 fragments and gel retardation assay

5 ng P-rC and 75 µg Nup153 fragments were incubated in transport buffer (20 mM HEPES pH 7.3/2 mM Mg acetate/5 mM Na acetate/110 mM K acetate/1 mM EGTA/2 mM DTT/ protease inhibitor [complete, Roche]) for 2 h at 37°C. 1.2×10^7^ anti-GST antibody (MoBiTec)-coated biomagnetic beads (Dynal) were added and incubated over night at 4°C.

The beads were washed 3× with transport buffer including one change of the cups. The beads were incubated for 1 min in PBS/0.1% NP-40, sedimented and washed 4× with PBS. The precipitated proteins were then separated on SDS PAGE and blotted to a PVDF membrane, which was saturated using 5% (w/v) milk powder/1×PBS prior to the addition of the first antibody (anti-GST antibodies, 1∶500, 2 h, RT). The membrane was washed 3×10 min in 1×PBS/0.1% Tween 20/0.5% milk powder and incubated with a peroxidase-labeled anti-rabbit antibody (1∶5000, 1 h, RT). The membrane was washed as described above, followed by an additional 15 min incubation in 1×PBS at RT prior to the visualization by film using ECL.

In gel retardation assays the incubation mixture of capsids and Nup153 fragments were separated on 0.7% agarose/TAE gels and blotted onto PVDF membranes by capillary blotting [71]. Nup-fragments were detected by anti-GST antibodies or and anti-His antibodies (dilution 1∶3000) as described above, HBV capsids were detected by anti-capsid antibodies (DAKO, 1∶10,000).

### Competition assays

Two hundred ng P-rC were incubated with 200 ng of GST-Nup153 and different concentrations of importin β for 2 h at 37°C in transport buffer. At the lowest concentration of importin β the ratio was 1 capsid : 28 GST-Nup153 : 33 importin β molecules. The capsids were precipitated by the addition of 1.2×10^7^ anti capsid antibody-saturated biomagnetic beads o.n. at 37°C. The beads were washed 3× with 0.1% BSA/PBS and once with 0.1% Nonidet P-40/PBS. The beads were transferred to a new cup and washed again 3× with PBS before the proteins were separated on a 3–8% SDS PAGE (Tris acetate gel). Co-precipitated GST-Nup153 was detected by Western blot using mAb414 as described above.

Alternatively 200 ng of capsids were bound to 1.2×10^7^ anti capsid antibody-saturated biomagnetic beads o.n. at 37°C followed by an incubation with 200 ng GST-Nup153 in transport buffer for 2 h at 37°C. After washing, varying amounts of importin β were added for 2 h at 37°C. The beads were then washed as described above and subjected to SDS PAGE and immune blot using mAb414.

### Microinjection into *Xenopus laevis* oocytes and electron microscopy

The cytosolic microinjection, preparation of the nuclei and electron microscopy are described elsewhere [72]. Six *Xenopus laevis* oocytes were used per sample. Per oocyte 1×10^7^ Mat-C or Mat-C UV were microinjected. P-rC, available in much higher concentrations than Mat-C, was microinjected in 2×10^8^ particles per oocyte. For quantification the number of NPC with and without capsids as well as their location at the pore were recorded in 50 nm sections.

### Nup153 silencing

HeLa cells were transfected with small interfering RNA (siRNA) (Dharmacon) against Nup153 at a final concentration of 10 nM using lipofectamine RNAiMAX (Invitrogen) according to the manufacture's instructions. The sequence used corresponded to nucleotide 2593–2615 of human Nup153 (AAGGCAGACUCUACCAAAUGUTT), which has been previously shown to accomplish efficient knock down of Nup153 in HeLa cells [10]. Expression of Nup153 was assessed by labeling Nup153 with a monoclonal antibody, SA1 [73]. Cells were analyzed by Western blot and used to assay nuclear import of P-rC two days post transfection. For control, mock transfection of cells without siRNA was performed. Visualization was performed using enhanced chemoluminescence and autoradiography films at different exposure times. The siRNA transfected cells were then used for transport assays after digitonin-permeabilization.

### *In vitro* transport and binding assays

To determine the nuclear import capacity, 25 ng of Mat-C or Mat-C UV were subjected to digitonin-permeabilized HuH-7 cells that were grown on 12 mm collagen-coated cover slips. Growth, permeabilization of the cells and indirect immune stain was described previously [19]. To allow comparison of the results the antibodies were the same as those used for the co-immune precipitations (anti-capsid [DAKO], mAb414). As secondary antibodies an Alexa488-labelled goat-anti-rabbit antibody (Invitrogen) and a Texas Red-labeled goat-anti-mouse antibody were added (Dianova). Microscopy was performed on a Leica DM IRBE confocal laser scan microscope using the filter settings for FITC, TRITC and Cy5 at a pinhole size of 1.

To study the effect of Nup153 capsid-interaction on the nuclear import of other substrates an *in vitro* transport assay was performed using a modified protocol of Kann et al. [18] In a first step after permeabilization and washing the NPCs were loaded with a geometrical dilution of P-rC (0, 25–12800 ng per 12 mm cover slip) for 30 min at 37°C in the presence of RRL, ATP and an ATP-generating system. Afterwards the cover slips were washed 3 times with 500 µl transport buffer/1×EDTA-free complete protease inhibitors/2 mM DTT to remove unbound capsids. In the last wash less than 3 pg of unbound P-rC were present (<300 pg per 0.5 ml washing solution of the last washing step, ∼5 µl remaining fluid estimated).

The conjugates (50 µg/ml) to be analyzed were added in a second transport reaction with new RRL, ATP and an ATP-generating system for 15 min at 37°C. For analysis of importin-mediated transport we used Alexa594-labelled BSA (Invitrogen) linked to the peptide PKKKRKVED that represents the NLS of the SV40T-Ag [74]. The import by transportin was analyzed using Alexa647-labelled BSA conjugated to the M9-domain of hnRNP A1 protein (YNNQSSNFGPMK) [75]. Both peptides were linked via a CGGG spacer to the BSA. Analysis of the conjugates by MALDI-TOF MS revealed a comparable conjugation of an average of 19 peptides per BSA molecule. Non-imported conjugates were removed by 3 washes in 500 µl transport buffer. Fixation, blocking reaction and immune stains of NPCs and capsids were done as described previously [18] using the mAB414 antibody or the anti-capsid antibody respectively. As second antibodies we used Alexa532-anti-rabbit and Alexa568-anti-mouse antibodies. Confocal laser scan microscopy was done using the TCS SP5 microscope (Leica) equipped with a HCX PLAPO 63×/1.4-0,6 Oil objective (Leica). Images were taken at a zoom of 2 with a size depicting ∼70 µm pinhole size.

### Quantification of nuclear import

The intranuclear area, flanked by the rim-like stain of the NPCs or P-rC stain was selected and the fluorescence signals of the imported cargos were determined (software LAS AF version 2.1.0). Calculations were done using Microsoft Excel. Although showing a large linear range absolute fluorophore numbers can be hardly obtained by fluorescence microscopy as the signal depends upon e.g. recording efficiency, filter setting, lens and amplification of the individual dye. To normalize the signals mean values of the positive import reactions were set as 100%. The mean values of nuclei recorded in the negative control at 4°C were set as 0% being only marginally above the background outside the permeabilized cells. Since the evaluated parameters (total nuclear import, concentration and cell size) showed a Gaussian normal distribution within each sample a comparison of the signals was done by Students' T-test.

### Quantification of nuclear binding of P-rC

HuH-7 cells (∼1×10^6^ cells) from confluent grown 10 cm dish were treated with trypsin, removed by pipetting in 15 ml D-MEM and sedimented at 4°C for 10 min at 200×g. The cells were resuspended in D-MEM and sedimented as described above. This step was repeated before the cells were resuspended in 5.5 ml D-MEM/40 µg/ml digitonin. After 5 min at 37°C permeabilization was controlled by microscopy of an aliquot. Cells were sedimented in aliquots that reflect the number of cells per cover slip in transport assays and resuspended in 0.5 ml transport buffer/2 mM DTT/PI. This sedimentation and resuspension was repeated twice. The permeabilized cells were resuspended in the transport mixture identical to that in the transport assays using cover slips containing different amounts of P-rC and incubated for 30 min at 37°C. After three washing steps by sedimentation and resuspension in transport buffer/2 mM DTT/1×EDTA-free complete protease inhibitors as above the permeabilized cells in an aliquot were counted. The amount of P-rC was determined as described above by native agarose gel electrophoresis and immune blotting.

### Quantification of NPCs

NPCs were quantified by comparing the amount of cellular Nup153 from digitonin-permeabilized cells with a standard dilution series of *E. coli*-expressed Nup153. Cells were grown and harvested and permeabilized as described for “Quantification of nuclear binding of P-rC”. Aliquots containing the same number of cells were denaturated and loaded on an 8% Tris-Glycin SDS PAGE (Anamed). Proteins were transferred onto a PVDF membrane and Nup153 was detected by mAb414 as described above.

## Supporting Information

Video S1 Capsid stain in HeLa cells transfected with mock-RNAi. Scan through the nuclei. The transfected cells were permeabilized using digitonin and incubated with P-rC in the presence of cytosolic proteins. The capsids were visualized by indirect immune fluorescence (green). The figure video demonstrates that all capsids remained bound to the nuclear envelope and did not enter the nucleus.
(7.96 MB AVI)

Video S2 Nup153 stain in same HeLa cells depicted in Video S1. Scan through the nuclei. The transfected cells were permeabilized using digitonin and incubated with P-rC in the presence of cytosolic proteins. Nup153 was visualized by indirect immune fluorescence (red).
(7.96 MB AVI)

Video S3 Capsid stain in HeLa cells transfected with Nup153-RNAi. Scan through the nuclei. The transfected cells were permeabilized using digitonin and incubated with P-rC in the presence of cytosolic proteins. The capsids were visualized by indirect immune fluorescence (green). The video shows that a significant proportion of capsids (green) entered the nucleoplasm but that the majority of capsids were retained at the nuclear envelope.
(8.12 MB AVI)

Video S4 Nup153 stain in same HeLa cells depicted in Video S3. Scan through the nuclei. The transfected cells were permeabilized using digitonin and incubated with P-rC in the presence of cytosolic proteins. Nup153 was visualized by indirect immune fluorescence (red). The video shows that the nuclei showed a significantly weaker Nup153 signal than the mock-RNAi transfected nuclei.
(8.12 MB AVI)
